# Seed‐Borne *Spirosoma pollinicola* in Commercial Hazelnuts: A Global Survey of Microbial Presence and Allergen Diversity

**DOI:** 10.1111/pce.70225

**Published:** 2025-10-06

**Authors:** Barbara Karpinska, Alessandro Fiocchi, Marta Biolatti, Ileana Manera, Christine Helen Foyer

**Affiliations:** ^1^ School of Biosciences University of Birmingham Birmingham UK; ^2^ Department of Pediatrics Division of Allergy, Pediatric Hospital Bambino Gesù IRCCS Vatican City Italy; ^3^ Soremartec Italia Srl – Technical & Scientific Direction Alba Italy

**Keywords:** hazelnuts, immune response, protein allergies, proteomics, seeds, skin prick test, Spirosoma

## Abstract

Serious allergic reactions are increasing globally. Within this context, fatal anaphylaxis from hazelnut allergies is a critical public health concern. Hazelnuts, which are a common ingredient of many foods, contain many proteins that cause severe allergic reactions. Hazelnuts from all of the major commercial growing locations worldwide contained *Spirosoma pollinicola* sp. proteins. This endotoxin‐producing bacterium is linked to the allergenicity of hazelnut pollen. We were unable to remove the contamination by *S. pollinicola* proteins, showing that this bacterium is a seed endosymbiont. Comparative proteomics revealed significant variations in the allergenic protein composition of nuts that correlated with patient immune responses. Hazelnuts from provenances 17 and 18 exhibited lower levels of key antigens, particularly Cor a 9 and Cor a 14, highlighting their potential as candidates for genetic modification to mitigate allergenicity. Moreover, Spirosoma protein persistence may influence hazelnut allergenicity and the patient's immune response.

## Summary Statement

Hazelnut protein allergies pose a significant health concern, yet progress in mitigating allergenicity is hindered by limited knowledge of the hazelnut microbiome and allergen variations between commercial cultivars. Deep‐shotgun discovery proteomics revealed the persistent presence of Spirosoma in the seeds of all cultivars tested, along with substantial variability in hazelnut allergenic proteins that is closely linked to the immune response in patients.

## 1 Introduction

Food allergies are a persistent and potentially life‐threatening health concern. The rising prevalence of food allergies in recent decades has led to significant clinical and societal challenges (Novembre et al. 2024). Public awareness of this issue is so acute that the recent approval of a new drug for food allergy reached the front page of the New York Times (https://www.nytimes.com/2024/02/25/health/children-food-allergies-xolair.html). Fatal reactions to milk, peanuts and tree nuts are well recognized; however, current gaps in understanding allergy pathogenesis hinder the ability to predict and prevent these events. (DuToit et al. 2024). Nuts, particularly tree nuts, are among the main causes of anaphylaxis in world populations and are ubiquitous in Western diets (Borres et al. 2022). There is currently no curative treatment for hazelnut allergy, and affected individuals must adhere to a restrictive diet and carry auto‐injective epinephrine. The burden of nut allergies extends beyond physical health, significantly impacting psychological well‐being by inducing stress and anxiety (Primeau et al. 2000). The rising prevalence of nut allergy further exacerbates these challenges, negatively affecting quality of life (Primeau et al. 2000; Avery et al. 2003; Arasi et al. 2023).

Hazelnuts (*Corylus* spp.) are a key ingredient in widely consumed foods, particularly chocolate‐based products, providing nutritional benefits and distinctive flavour. However, hazelnuts are also among the most potent food allergens, representing the leading cause of nut sensitization in general populations (Giannetti et al. 2023). The prevalence of hazelnut allergy is estimated at 0.86% in European adults, 0.28% in European children, and 0.60% in the American and Australian populations (Calamelli et al. 2021). Hazelnut allergy is the fourth leading cause of food anaphylaxis in Europe, producing a wide array of symptoms, particularly in patients who are sensitized to highly stable allergens, such as seed storage proteins (Geiselhart et al. 2018). Compared to other types of food allergies, allergies to tree nuts, including hazelnuts, tend to persist throughout life, typically emerging between the ages of 2 and 5 years. Allergic patients may react even to small quantities of food containing hazelnuts, with an eliciting threshold that is amongst the lowest in the field of food allergens. This is due to the extreme allergenic power of some of the hazelnut proteins, which are structural antigens (Hartz et al. 2010).

Belonging to the Betulaceae family, hazelnut is a monoecious, self‐incompatible wind‐pollinated broadleaf species that typically thrives as an understorey shrub alongside birches and alders. The sporophytic self‐incompatibility of the species is under the control of a single locus with multiple alleles (Bassil and Azarenko 2001). Hazelnut cultivation has expanded globally in response to increasing industrial demand. The trees are generally propagated vegetatively via shoot or root cuttings to maintain genetic uniformity and reduce propagation times. *Corylus avellana* L., the European hazelnut, and *Corylus colurna* L., the Turkish hazelnut, are widely grown in Europe and are important genetic resources for breeding programs.

Seeds from ecologically and geographically diverse plants can harbour a characteristic range of microbiota, which are often identified by amplification and sequencing of genetic fragments. Epiphytic microbiota can consist of synergistic, commensal, and potentially pathogenic microbes that play a crucial role in health and susceptibility to disease. Such microbiota that often reside on seed surfaces play a functional role in plant growth, development, and seed storage, with seed‐borne endosymbionts contributing to microbial populations within other plant tissues. Previous studies have identified *Spirosoma pollinicola* sp. nov. in the pollen of the European hazelnut (Ambika Manirajan et al. 2018). This endotoxin‐producing bacterium is thought to contribute to the high allergenicity of hazelnut pollen, leading to the release of both chemokines and cytokines from epithelial A549 cells (Ambika Manirajan et al. 2022). Pollen‐associated bacteria may be transferred to seeds during pollination. Recent studies suggest that this allows the transmission of the plant microbiome across generations through the pollen grain, which acts as a vector for bacterial transfer to the developing seed. However, the potential impact of microbiome transfer on hazelnut allergenicity is currently unknown.

Genomic data and proteomic data provide valuable insights for hazelnut breeding, particularly in an effort to reduce allergenic protein content. RNA‐seq characterization of complementary DNA libraries from four hazelnut tissues, including leaves, catkins, bark and whole seedlings, led to the assembly of a 6.8 Gb hazelnut transcriptome (Rowley et al. 2012). De novo transcriptome assembly of *C. avellana* cv. Tombul and *C. colurna* identified 70 265 and 88 343 unigenes, respectively (Ulu et al. 2023). Transcriptome data, whole‐genome re‐sequencing, and ChIP sequencing and characterization of differentially expressed genes in the hazel genome have been applied to investigate the regulation of ovule and nut development (Cheng et al.^2024^). However, relatively few studies have explored the genetic variation and control of hazelnut allergens. A study involving 12 groups of allergenic proteins and 13 hazelnut varieties revealed that all samples had similar IgE‐reactivity profiles (Ribeiro et al. 2020). Ten recognized hazelnut allergens are proteins (or glycoproteins): Cor a 1, Cor a 2, Cor a 8, Cor a 9, Cor a 10, Cor a 11, Cor a 12, Cor a 13, Cor a 14 and Cor a 15 (Supporting Information S4: Table S1). Of these, Cor a 9, an 11S seed‐storage globulin (legumin), Cor a 14, a 2S albumin, and – to a lesser extent – Cor a 8, a nonspecific lipid transfer protein (LPT), are associated with the most severe allergic reactions (Caffarelli et al. 2021). However, the genetic variability in the expression of these proteins in hazelnuts remains poorly studied, and little information is available on how their abundance varies across different cultivars, particularly in response to environmental conditions. The following study was therefore conducted to investigate the extent of natural environmental and genetic variation in the hazelnut allergen profile. The comparative proteomics analysis reported here reveals significant variations in allergenic protein composition among major commercial hazelnut‐growing areas. These variations correlated with patient immune responses, as evidenced by the skin prick test results, underscoring the importance of cultivar selection in allergen management strategies. Surprisingly, we detected Spirosoma proteins in all nut samples, suggesting that this endosymbiont may influence hazelnut allergenicity.

## 2 Material and Methods

Hazelnut samples used in this study come from different parts of the world, covering the main cultivation areas: Turkey, Italy, Chile, Azerbaijan and the United States. The nuts were sorted, cracked, shelled, and calibrated before shipping, with stringent quality checks conducted in the same manner as they would be for roasting, processing and consumption.

### 2.1 Preparation of Defatted Flour

Raw hazelnuts were subjected to five cycles of grinding for 2 min each to achieve a fine texture. Following grinding, hazelnut flour was homogenized into a fine powder using a mortar and pestle to ensure uniformity and the smallest particle size. To prepare defatted hazelnut flour, 1 g of hazelnut powder was extracted with five volumes of *n*‐hexane, followed by a final defatting step using five volumes of acetone. Extractions were performed at 4°C, followed by 30 min of sedimentation and filtration. After defatting, hazelnut flour was air‐dried under a fume hood to remove any residual solvent.

### 2.2 Protein Quality Assessment

Samples (100 mg) of defatted flour were extracted with 1.5 mL of sodium dodecyl sulphate (SDS) buffer (pH = 6.8) as described by de Angelis et al. (2022), with some modifications. Each sample was heated at 40°C for 20 min on a thermo‐shaker (Thermomixer Comfort, Eppendorf‐Netheler‐Hinz GmbH, Hamburg) with a stirring function (1000 rpm), and then centrifuged. Supernatants were precipitated with cold 20% TCA/acetone. Pellet was subsequently washed with acetone twice and dissolved in 100 μL of Urea buffer (7 M Urea, 0.1 M Tris pH = 8, 0.001 M EDTA and 1% DTT), and used for protein estimation by Bradford. 15 ug of proteins were later separated on SDS PAGE acrylamide gradient gels (Any kD Mini‐PROTEAN TGX Precast Protein Gels). The remaining powder was subsequently used for shotgun proteome analysis.

The protein extraction and all procedures for mass spectroscopy were performed by Biogenity ApS, Aalborg, Denmark, as follows. Powdered hazelnut samples were transferred to 1.5 mL LoBind Eppendorf tubes containing 100 µL of lysis buffer (consisting of 6 M Guanidinium Hydrochloride, 10 mM TCEP, 40 mM CAA, 50 mM HEPES, pH = 8.5) and 3 mm tungsten carbide beads (Qiagen). Samples were processed twice with the TissueLyser II (Qiagen), and 30 µg of sample was diluted to 20 µL with lysis buffer and taken forward for digestion and TMT labelling with 16plex tags (Thermo). Peptides were eluted over a 44‐min gradient on a 15 cm PepSep Endurance column (Bruker), and analyzed with an Orbitrap EclipseTM TribridTM instrument (Thermo Fisher Scientific) with FAIMS ProTM Interface (ThermoFisher Scientific) switched between CVs of −50 and −70 V with cycle times of respectively 2 and 1.5 s. Full MS spectra were collected at a resolution of 120 000, with normalized AGC target set to ‘standard’ or maximum injection time of 50 ms and a scan range of 375–1500 *m/z*. MS1 precursors with an intensity of > 5 × 10^3^ and charge state of 2–7 were selected for MS2 analysis. Dynamic exclusion was set to 120 s, the exclusion list was shared between CV values, and Advanced Peak Determination was set to ‘off’. The precursor fit threshold was set to 70% with a fit window of 0.7 *m/z* for MS2. Precursors selected for MS2 were isolated in the quadrupole with a 0.7 *m/z* window. Ions were collected for a maximum injection time of 50 ms, and the normalized AGC target was set to ‘standard’. Fragmentation was performed with a CID normalized collision energy of 30% and MS2 spectra were acquired in the IT at a rapid scan rate. Precursors were subsequently filtered with an isobaric tag loss exclusion of TMT and precursor ion exclusion set to 25 ppm low and 25 ppm high. Precursors were isolated for an MS3 scan using the quadrupole with a 2 *m/z* window, and ions were collected for a maximum injection time of 86 ms and a normalized AGC target of 300%. Turbo TMT was deactivated, and the number of dependent scans was set to 5. Isolated precursors were fragmented again with 50% normalized HCD collision energy, and MS3 spectra were acquired in the Orbitrap at 50 000 resolution with a scan range of 100–500 *m/z*. MS performance was verified for consistency by running a complex cell lysate quality control standard. The raw files were analyzed using Proteome Discoverer 2.4 (Thermo Fisher Scientific). TMT reporter ion quantitation was enabled in the processing and consensus steps, and spectra were matched against the *C. avellana* obtained from UniProt. Dynamic modifications were set as Oxidation (M), Deamidation (N, Q) and Acetyl on protein N‐termini. Cysteine carbamidomethyl (on C residues) and TMT 16‐plex (on peptide N‐termini and K residues) were set as static modifications.

### 2.3 The Data Analysis

The data were filtered to include only proteins with at least two unique peptides, 10% sequence coverage, and a minimum sequence score of 30. Data analysis was carried out using Perseus version 2.0.7 software (https://maxquant.net/perseus/). The data set was filtered to retain proteins with at least 60% valid values across all samples, or at least 75% within each experimental group, and a cutoff of 100 for normalized intensity. The distribution of log_2_ ratio values of filtered data was analyzed as a frequency histogram. The data were symmetrically distributed and used for downstream analysis. Multi‐sample ANOVA and two‐sample *t*‐tests were performed using Perseus with default settings (S0 = 0.1, false discovery rate = 0.05).

Proteomics data are available in the ProteomeXchange Consortium via the PRIDE partner repository with the data set identifier PXD052613 and 10.6019/PXD052613. (https://www.ebi.ac.uk/pride/review-dataset/1f7ba346d43f47b3a38f492d78ffa5e5, Project accession: PXD052613).

### 2.4 Functional Categories of Hazelnut Proteins by Gene Ontology

The analysis of protein functional profiles and KEGG pathways was based on the set of 372 proteins identified in all hazelnut samples. ID mapping of UniProt database (Release 2014_02) was used to find protein GO terms. As a result, proteins were annotated with corresponding GO numbers and then classified into biological process (BP), cellular component (CC) and molecular function (MF) using ShinyGo 0.80 and g: Profiler.

### 2.5 Spirosoma Proteins

Whole nuts were washed in water at 40°C for 5 min and then with ethanol. This procedure was repeated three times before the samples were extracted for proteomics analysis. In addition, other samples were soaked in bleach (10%) for 5 min before extraction for proteomics analysis. The proteomics analysis was performed as described for the shelled hazelnuts, except that proteins were identified using the UniProt database.

### 2.6 Skin Prick Tests

Skin prick tests were performed on 31 children, aged between 4 and 14 years (with an equal distribution of males and females and oral food challenge) with confirmed hazelnut allergies. The number of patients is consistent with the established minimum number that is necessary for inferences on sensitization and reactions in food allergy studies, which is 29 (Klein Entink et al. 2014). The sample size used in the present study is thus suitable for fitting sensitization distributions in food allergy. With this data, it is also possible to evaluate the threshold reactivity using a parametric model on interval‐censored failure time data, based on the individual NOAEL and lowest LOAEL (Valluzzi et al. 2025). Data obtained from this number of patients is also used for the evaluation of hypoallergenic products (Dahdah et al. 2022). All tests were performed with ground hazelnut samples from 1 to 18 cultivars mixed with 0.9% saline. Samples were tested on the patients in duplicate. The appearance of a wheal with a mean diameter of at least 3 mm was considered a positive response. Skin reactivity was expressed by measuring the area of the wheals (Heinzerling et al. 2013).

## 3 Results

### 3.1 Detection of Spirosoma

**Figure 1:**
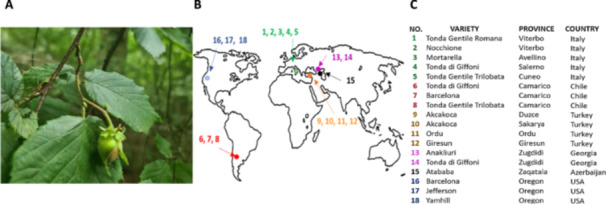
An image of a typical hazelnut developing a tree in the United Kingdom (A); hazelnut production areas (B) and geographical locations (C).

**Figure 2:**
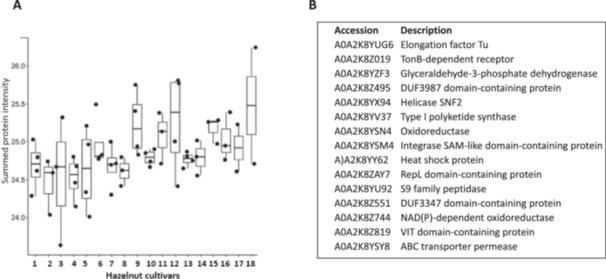
Box plot of summed intensities of *Spirosoma pollinicola* proteins in all hazelnut cultivars (1–18). The plot shows the distribution of summed protein intensities across all 18 cultivars. Data are based on four biological replicates. Boxes represent the interquartile range (IQR), horizontal lines within boxes indicate the median, and dots represent outliers. Statistical analysis revealed no significant differences among cultivars (A). Set of proteins identified in samples 1–18 (B).

Hazelnut samples were collected from 18 global commercial plantations (Figure 1). The proteomics analysis undertaken in these studies revealed the presence of Spirosoma in seeds from all the growing commercial regions (Figure 2A). The bacterial proteins identified in hazelnut seeds included numerous metabolic enzymes (Figure 2B), suggesting that the bacteria remain viable within the seeds. Moreover, extensive washing or sterilization of the seed coat failed to remove these proteins, indicating a stable association with the seed microbiome. Sequence comparisons confirmed that the proteins shown in Figure 2 arise from *S. pollinicola* sp. Nov, which has previously been identified through genome‐based comparisons between HA7^T^ and *Spirosoma linguale* DSM 74^T^ and *Spirosoma fluviale* DSM 29961^T^ (Ambika Manirajan et al. 2022).

### 3.2 The Hazelnut Proteome

**Figure 3:**
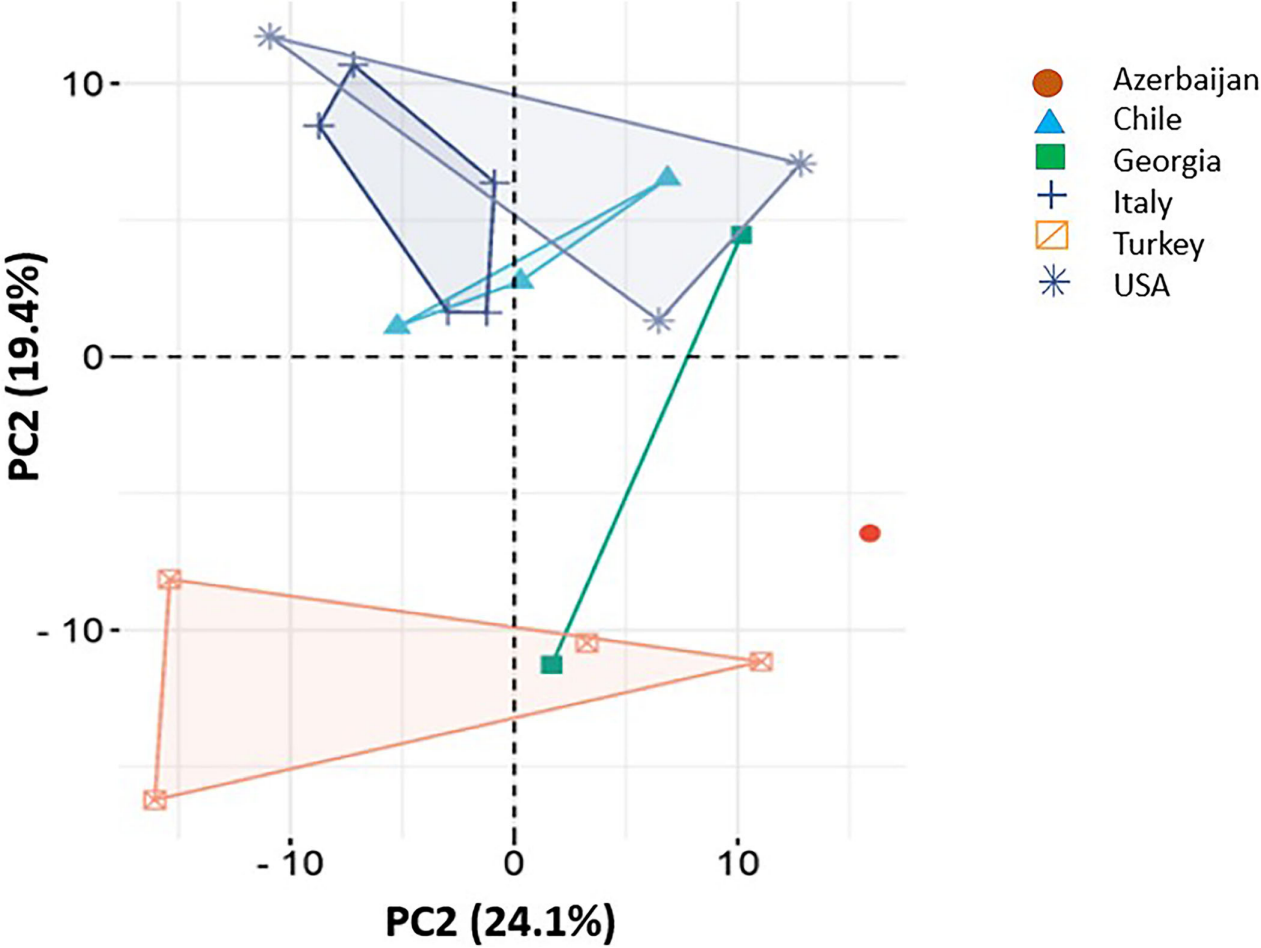
Principal component analysis (PCA) between samples and groups of hazelnuts. Five country groups are represented by different colours: Azerbaijan (1 variety), Chile (3 varieties), Georgia (2 varieties), Italy (5 varieties), Turkey (3 varieties) and the United States (3 varieties). Varieties are represented by dots of different shapes. [Color figure can be viewed at wileyonlinelibrary.com]

Shotgun proteomics enabled the comprehensive profiling of the hazelnut proteome across commercial cultivars (Supporting Information S1: Figure 1). Alignment of peptide sequences with established databases, including UniProt, Allergome, NCBI and Ensembl Plants. These databases were used to identify all of the known hazelnut allergens and their variants. Principal component analysis (Figure 3) revealed that the hazelnut proteomes were variable and that they were grouped according to the geographic location of origin. Tonda di Giffoni trees are cultivated in multiple regions (Italy, Chile and Georgia; locations 4, 6 and 14, respectively), while Tonda Gentile Trilobata is grown in Italy and Chile (locations 5 and 8, respectively). The Barcelona variety is grown in Chile and the United States (locations 7 and 16, respectively). Some samples from different sources clustered more closely than others (Figure 3, circles red and square green), which may reflect regional influences on the proteome composition.

**Figure 4:**
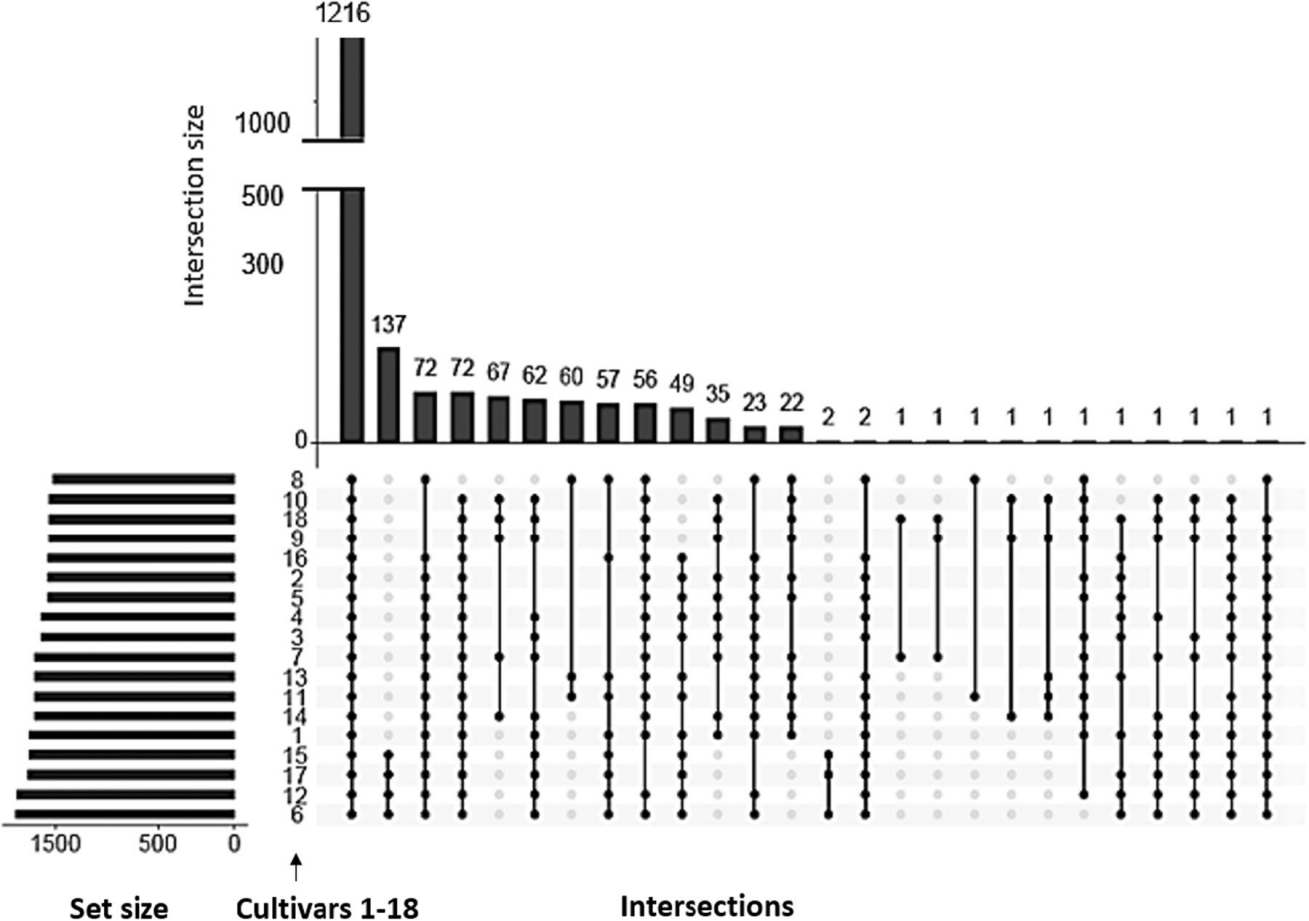
UpSet diagram illustrating intersections among protein sets from 18 cultivars. Horizontal bars indicate the total number of proteins in each cultivar, while vertical bars show the size of protein intersections (proteins found in the exact combination of cultivars indicated by the filled dots below). Among the 1953 identified proteins, 637 proteins were present only in specific cultivar combinations (25 intersections), while 1216 proteins were shared by all cultivars.

Of the 1953 proteins that were identified overall in the hazelnut proteome, 1216 proteins were common to all samples, and 637 proteins were detected only in specific cultivar combinations (Supporting Information S1: Figure 1). Moreover, 140 unique proteins were identified in one or more samples (Figure 4). However, the normalized intensity values of many of these proteins were below the cutoff threshold of 100. They were therefore excluded from further analysis. Of the 1216 proteins that were found in all samples, 621 were subjected to further analysis. A significant differential abundance was found for 372 proteins (Figure 4).

**Figure 5:**
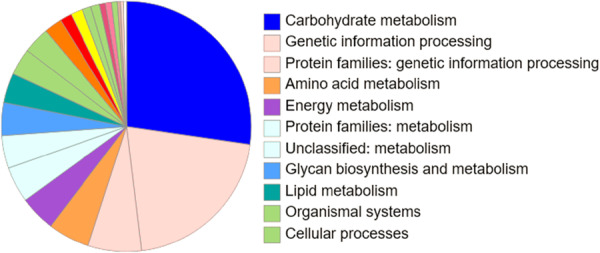
Pie chart showing the functional distribution of the 372 proteins that were assigned to pathways, of which 262 had corresponding KEGG Orthology (KO) numbers, indicating that not all identified proteins are associated with defined metabolic pathways. KEGG Orthology numbers were assigned using BlastKOALA (https://www.kegg.jp/blastkoala). The three largest categories are: 26% of proteins classified under carbohydrate metabolism; 19% of proteins involved were classified as involved in pathways related to Genetic information processing, and 8% of proteins were assigned as associated with Protein information processing. All proteins with assigned KO numbers are listed in Supporting Information S5: Table 2. [Color figure can be viewed at wileyonlinelibrary.com]

Functional enrichment of gene ontology terms for the nut proteome (Supporting Information S2: Figure 2) identified 424 proteins that fell into the category of MF, 809 in the category of BPs and 169 in the category of CCs (Supporting Information S2: Figure 2). The Gene Ontology (A, GO) function enrichment analysis of the hazelnut proteome (Supporting Information S3: Figure 3) revealed that 371 proteins were statistically different in terms of abundance. Many of these differentially abundant proteins are known to be involved in metabolism, such as carbohydrate storage and turnover (e.g., amylase, starch synthase) and stress tolerance and defence (e.g., superoxide dismutase), as well as other important cellular functions (Supporting Information S3: Figure 3). Of the 212 proteins within the MF category (Supporting Information S3: Figure 3), most were designated as having catalytic functions (GO:0140096), hydrolase activity (GO:0008152), ATP binding (GO:0005524) and peroxidase activity (GO:0004601), and also carbohydrate binding (GO:0030246). The functions of the 84 proteins that were placed in the category of BPs include phosphorus metabolism (GO:0006793), response to stimulus (GO:0050896) and transport (GO:0006810). Only 25 proteins were classified within the GO term CCs, which allocates proteins according to their localization in the intercellular (cell wall/apoplast) and intracellular compartments. These include proteins located in the apoplast (GO:0048046) and cytoplasm (GO:0005737), as well as those localized in membrane complexes (GO:0098796) and ribosomes (GO:00057840). The pathway enrichment analysis assigned 372 differentially abundant proteins to pathways (Figure 5). Many of the differentially expressed proteins in the hazelnut proteome were involved in carbohydrate metabolism, as well as nutrient reserve and energy metabolism, particularly involving malate, serine and glucose 6‐phosphate pathways (Figure 5). The KEGG IDs of these proteins can be found in Supporting Information S5: Table 2. In addition, Supporting Information S6: Table 3, shows the top 10 enriched GO IDs for three GO categories (BP, MF and CC).

### 3.3 Allergen Identification

Hazelnut allergens were identified based on the World Health Organization (WHO) and the International Union of Immunological Societies (IUIS) Allergen Nomenclature databases, alongside the Allergome lists of *C. avellana* allergens and iso‐allergens (Supporting Information S4: Table S1). The detected allergens include Cor a 1 (Bet v 1 homologue), Cor a 2 (profilin), Cor a 8 (LPT), Cor a 9 (11S legumin), Cor a 11 (7S vicilin), Cor a 14 (2S albumin) and the oleosins Cor a 12, Cor a 13 (oleosin protein types) and Cor a 15 (7S vicilin‐type seed storage protein), Cor a 16 (2S albumin) and Cor a thaumatin‐like protein (TLP) (https://www.allergome.org/script/search_step2.php). Stringent selection criteria were applied to ensure reliable and significant allergen identification. This included a minimum sequence coverage of 10%, at least two unique peptides, and a sequence score higher than 30. One exception that had a lower coverage (at 12%) was Cor a 16. However, the high number of unique peptides associated with it provided confidence in the correct identification of this allergen. A second exception is the minor allergen Cor a TLP, which is recognized by less than 10% of patients who are allergic to hazelnuts (Palacín et al. 2012). In the present analysis, this protein was identified with low confidence, based only on 1 unique peptide, and with 6% coverage by all identified peptides. It was therefore not subjected to further analysis (Supporting Information S4: Table S1).

### 3.4 Iso‐Allergens and Allergen Variants

Most allergens, including Cor a 6, Cor a 8, Cor a 10, Cor a 13, Cor a 14 and Cor a 15, were represented by only one isoform, as identified by a single variant (Supporting Information S4: Table S1). In contrast, Cor a 1 was represented by multiple isoforms (Cor a 1.0101 to Cor a 1.0801: Allergome database). Three isoforms were identified in the 18 samples: Cor a 1.0401, Cor a 1.0402 and Cor a 1.0501. Some allergens, including Cor a 2, Cor a 9 and Cor a 11, were represented by two or more isoforms: Cor a 2, Cor a 2.0101; Cor a 9, Cor a 9.0101, and Cor a 11.0101, Cor a 11.0102, respectively. Each pair of isoforms exhibited a high degree of homology (98%), in which sequences differed only in a few amino acids. Cor a 2 and Cor a 11 are encoded by a single gene, while the two isoforms of Cor a 9 are encoded by two different genes. Of the three Cor 16 isoforms listed in the Allergome database, only one isoform was detected in the hazelnut samples tested, subjected to the present analysis (Supporting Information S4: Table S1).

### 3.5 Cultivar‐Dependent Variations in Allergen Composition

**Figure 6:**
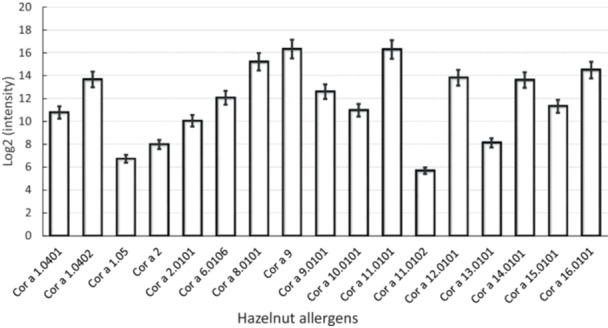
The relative abundance of each identified allergen across 18 hazelnut cultivars. Abundance is expressed as the mean log_2_ intensity across all cultivars and their four biological replicates. Error bars represent the standard error of the mean. All the allergens are listed in Table 1.

The Cor a 9 and Cor a 11 proteins were more abundant than other allergens in all samples, while Cor a 1.05, Cor a 2, Cor a 11.0102 and Cor a 13.0101 were the lowest in abundance (Figure 6). Significant differences in the relative abundance of the Cor a 1, Cor a 2, Cor a 9, Cor a 11 and Cor a 12 isoforms were observed, with Cor a 11 being the most abundant (Figure 6). Levels of Cor a 11.0101 were 60% higher than Cor a 11.0102 (Figure 6). Cor a 8, 9, 11 and 14 accounted for only about 1%–4% of the total nut protein (Figure 6).

**Figure 7:**
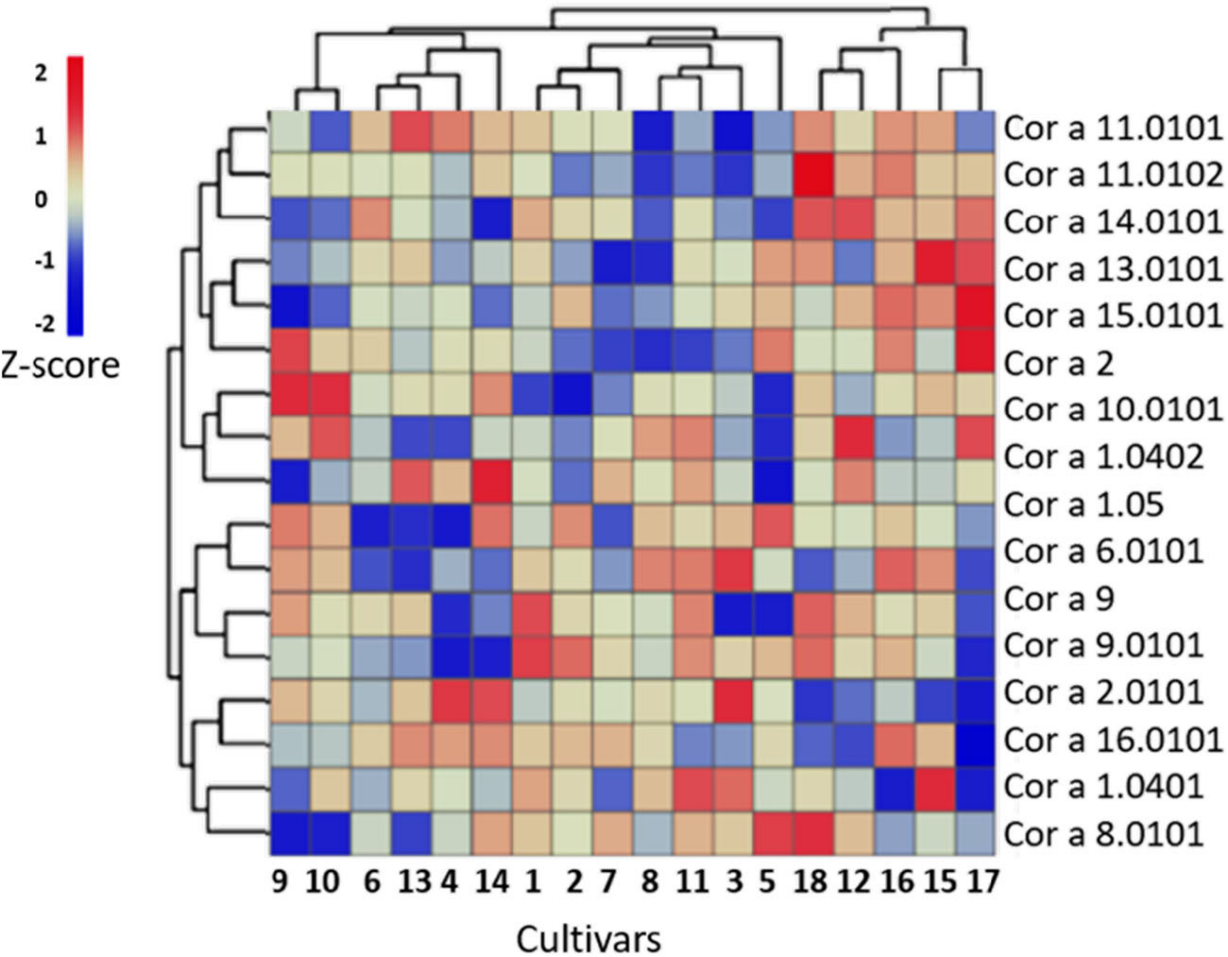
Hierarchical clustering of the allergenic proteins in all 18 hazelnut cultivars. Colours represent the log_2_‐transformed average protein intensities normalized by *z*‐score. The colour intensity represents the log_2_‐transformed average protein intensities, which were normalized using *z*‐score, with more abundant proteins shown in red and less abundant proteins shown in blue. [Color figure can be viewed at wileyonlinelibrary.com]

**Figure 8:**
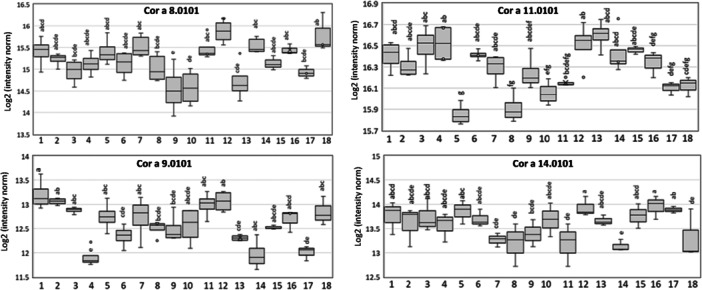
Box plots showing the log₂ protein intensity of four proteins that are associated with severe allergic reactions in all 18 cultivars. Boxes represent the interquartile range, the central line indicates the median, and whiskers show variability. Different letters indicate significant differences (one‐way ANOVA with Tukey's post hoc test, false discovery rate < 0.05).

Hierarchical clustering of the nut allergens revealed significant differences in the abundance of the proteins between some of the samples (Figure 7). Pairwise comparisons identified significant differences in the levels of major allergens between two or more cultivars. For simplicity, the variations in relative abundance between samples are shown only for the four main allergens (Figure 8). For example, the levels of Cor a 9.0101 were significantly lower in samples 4, 14 and 17, while Cor a11 levels were significantly lower in samples 5 and 8. Conversely, Cor a 8 iso‐forms were most abundant in samples 12 and 18 (Figure 8).

### 3.6 Variations in Immune Responses to Hazelnut Cultivars

**Figure 9:**
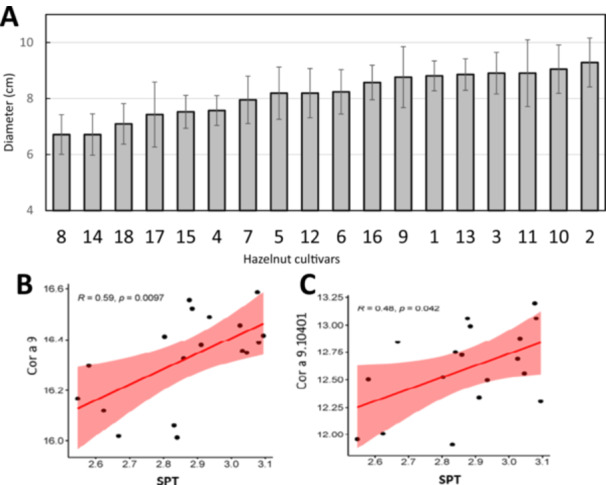
Skin prick test analysis, showing the average wheal diameter of the immunological response (A); The Pearson correlation coefficient (*r*) showing the correlation between allergen abundance and mean wheal size in the skin prick test (B). Correlation plots for Cor a 9.0101 (B) and Cor a 9 (C): *p*‐values 0.0097 and 0.042, respectively. [Color figure can be viewed at wileyonlinelibrary.com]

Skin prick test analysis on patients with hazelnut allergies revealed variations in the average wheal sizes and hence responses to the nut extracts from the 18 samples of 6 growing areas: Azerbaijan, Chile, Georgia, Italy, Turkey and the United States (Figure 9a). Weaker immune responses were observed in cultivars 8, 18 and 14, which had lower levels of Cor a 9.0101 and Cor a 11 levels than other samples, compared to other cultivars. In contrast, cultivars 2, 10 and 11 showed much stronger responses (Figure 9A). Relationships between the skin prick tests and allergen levels between the 18 growing regions were further analyzed using the Pearson correlation statistic method. For most of the allergens, the correlations were weak or negligible (*R*_s_ range −0.22 to 0.34, *p*‐value range 0.04 to 0.86 L, Figure 9B,C). However, a positive statistically significant correlation was observed for Cor a 9 (*R*_s_ = 0.59; *p*‐value = 0.0097) and Cor a 9.0101 (*R*_s_ = 0.48; *p*‐value = 0.042) (Figure 9A–C).

**Table 1:** Hazelnut allergens.

Accession	Name	Coverage (%)	Peptides	Unique peptides	# AAs	MW (kDa)	Score
Q9SWR4	Cor a 1.0401	81	11	6	161	17.6	924.18
Q9FPK4	Cor a 1.0402	81	12	7	161	17.5	1133.24
A0AA49XEK8	Cor a 1.05	15	2	2	139	15.5	59.31
Q9AXH4	Cor a 2	37	4	3	131	14.1	203.14
Q9AXH5	Cor a 2.0101	58	4	3	115	12.7	122.09
A0A0U1VZC8	Cor a 6.0101	44	11	11	308	34.3	532.77
Q9ATH2	Cor a 8.0101	67	8	5	115	11.8	1859.74
A0A0A0P7E3	Cor a 9	63	33	10	514	58.8	11 061.16
Q8W1C2	Cor a 9.0101	64	30	3	515	59.1	10 394.41
Q9FSY7	Cor a 10.0101	30	17	10	668	73.5	548.97
Q8S4P9	Cor a 11.0101	75	27	8	448	50.8	6939.57
UTE00285	Cor a 11.0102	58	21	2	469	53.1	4482.88
Q84T21	Cor a 12.0101	38	6	6	159	16.7	1064.85
Q84T91	Cor a 13.0101	26	3	3	140	14.7	205.14
D0PWG2	Cor a 14.0101	52	7	5	147	17.1	2360.38
C0HM28	Cor a 15.0102	44	7	6	169	17.7	724.73
UTE00286	Cor a 16.0101	12	14	14	1216	137.9	2438.17

## 4 Discussion

The global hazelnut supply chain requires high‐quality supplies of fresh hazelnuts throughout the year. In addition to existing cultivation areas, new plantations are emerging in countries such as Chile, Oregon, Georgia and the Balkans. Nuts from all commercial sources contained Spirosoma as an endosymbiont, suggesting a universal nature of the relationship between the bacteria and the nuts. Our findings provide the first evidence that all commercial sources of hazelnuts contain Spirosoma as an endosymbiont that is likely to be transferred through the pollen. While such microbiota often reside on the seed surfaces and are often identified by amplification and sequencing of genetic fragments, the data presented here reveal the presence of Spirosoma proteins within the nuts, because they were not removed by repeated washing or sterilization. Although the functional significance of the presence of Spirosoma proteins remains unclear, it is possible that their presence may influence the overall protein composition of the hazel nut seeds. This finding lays the foundations for further investigations into the interactions between the microsymbiont and its host that influence nut allergen contents. Further microbiological and immunological studies are needed to determine the role of Spirosoma in the allergenicity of hazelnuts. The observed genotypic variation in protein composition revealed in the present study provides novel insights into genotype/environment interactions that influence the nut proteome. We have identified small but important variations in the protein composition of hazelnuts from the different commercial provenances. While processing can significantly decrease the levels of individual nut allergens (Cuadrado et al. 2021), the protein composition of the nuts is inherently dependent on genetic components and their regulation.

Proteomics approaches have previously been used to analyze the effects of processing in reducing the allergenicity of hazelnut proteins (De Angelis et al. 2022) and evaluate the allergenic potential of 13 genetically diverse sets of hazelnuts (Ribeiro et al. 2020). Interestingly, despite the genetic diversity of hazelnut varieties and possible variety‐dependent differences in the IgE‐binding properties, only minor differences were found at the level of genes encoding Cor a 8, Cor a 9 and Cor a 14 in the 13 hazelnut varieties tested (Ribeiro et al. 2020). The shotgun proteomics approach undertaken in the present study would largely support this conclusion, however small but significant differences in the levels of major allergens were revealed among cultivars. Moreover, principal component analysis revealed significant proteome variations associated with both geographical origin and genetic background. Notably, the levels of some allergens, such as Cor a 9.0101, were significantly lower in some samples (4, 14 and 17), while Cor a 8 iso‐forms were most abundant in samples 12 and 18 (Figure 7). Further research is required to determine the full significance of such differences for the overall allergenicity of the hazelnuts.

In the interests of time, this study did not focus on all 1216 proteins present in all nut samples, or the unique proteins that were identified in only one or more of the samples. However, a future analysis of these data could be useful in revealing the molecular and metabolic mechanisms that underpin the observed differences in allergen levels. Importantly, our study found that the variations in the accumulation of allergens correlated with variations in the immune reactions among patients. The skin prick tests reported here revealed variations in the intensity of immunological reactions that correlated with the abundance of both Cor a 9 iso‐allergen. Given the semi‐quantitative nature of the measurement of this parameter, further studies involving larger patient cohorts are required to fully explore the relationships between the levels of allergens in different samples and the intensity of immune reactions.

The data presented here have identified provenances 17 and 18 as having lower levels of key antigens, making them prime candidates for genetic interventions aimed at decreasing the levels of specific antigens. This is a crucial next step in plant allergen science because many of the major allergens in hazelnuts and other tree seeds are heat‐stable and hence highly resistant to degradation during processing. Hazelnut in vitro cultures are used for commercial production of secondary metabolites such as paclitaxel (Gallego et al. 2017). In addition, hazel plantlets can be regenerated from callus tissues by differentiation induced by exogenous growth regulators (Salehi et al. 2017). It should therefore be possible to ectopically express transcription factors that regulate totipotency and stimulate cell proliferation (Jha and Kumar 2018), while silencing other transcription factors to stimulate plantlet formation and accelerate flowering. CRISPR‐CAS technology can then be used to decrease the expression of specific allergenic proteins in the hazelnut seeds.

Finally, our findings confirm that hazelnut seeds from ecologically and geographically diverse commercial trees can harbour *S. pollinicola*. Although the functional significance of this endosymbiont for the growth and development of the trees is unknown, this transfer facilitates the presence of *S. pollinicola* in the pollen of the next generation. Hence, the effective removal of the bacteria from the pollen would require a stringent endosymbiont elimination from the seeds. A deeper understanding of the seed microbiome is thus essential in devising strategies to mitigate allergenicity of plant organs.

## Conflicts of Interest

The authors declare no conflicts of interest.

## Supporting information


**Supplemental Figure 1:** Hierarchical clustering of the 1,941 proteins identified in the 18 cultivars (four replicates each). Colours show log₂ protein intensity normalized by z‐score (blue: low abundance; red: high abundance).

**Supplemental Figure 2:** Functional enrichment analysis of proteins identified in the whole proteome. Gene Ontology (GO) terms for Molecular Function (MF), Biological Process (BP), and Cellular Component (CC). After filtering based on protein intensity, consistency across replicates, and GO annotation availability, 1,402 proteins were included in GO enrichment analysis. A total of 424, 809, and 169 GO terms are presented for MF, BP, and CC, respectively. Functional enrichment was performed using g:Profiler (https://biit.cs.ut.ee/gprofiler/). Each filled circle represents a specific GO term within the respective category. Numbers associated with the dots indicate the top enriched GO terms which are listed in Supplemental Table 3. P values were adjusted for multiple testing using the Benjamini‐Hochberg method (Padj).

**Supplemental Figure 3:** Gene Ontology (A, GO) function enrichment analysis of the hazelnut proteome, in which 371 proteins had statistically significant changes in protein abundance. GO categories: molecular function (MF), biological processes (BP) and cellular component (CC). The analysis included 371 differentially expressed proteins. X axis represents GO function (three categories: BP‐biological processes, MF‐ molecular function, CC‐ cellular components).

**Supplemental Table S1:** List of major hazelnut allergens detected in the commercial samples.

**Supplemental Table S2:** KEGG pathway assignment for 356 proteins. Of these, the 16 allergenic proteins (listed in Table 1) were excluded because they are not classified as metabolic proteins and lack KEGG Orthology numbers.

**Supplemental Table S3:** Top ten GO terms across all three categories (BP, MF, CC) showing significant enrichment. Adjusted p‐values were calculated using the Benjamini‐Hochberg (B‐H) method. Enrichment fold (EF) was calculated as the ratio of the proportion of queries annotated to the term to the proportion of background genes annotated to the term.

## Data Availability

Proteomics data are available in the ProteomeXchange Consortium via the PRIDE partner repository with the data set identifier PXD052613 and 10.6019/PXD052613. (https://www.ebi.ac.uk/pride/review-dataset/1f7ba346d43f47b3a38f492d78ffa5e5, Project accession: PXD052613).
