# Supplementary material for: Seed‐Borne Spirosoma pollinicola in Commercial Hazelnuts: A Global Survey of Microbial Presence and Allergen Diversity
Source: Plant Cell Environ. 2025 Oct 6;49(1):398–409. doi: 10.1111/pce.70225 (PMC12675980; doi:10.1111/pce.70225)
Supplement: Supplementary file 1 — Supplemental Figure 1: Hierarchical clustering of the 1,941 proteins identified in the 18 cultivars (four replicates each). Colours show log₂ protein intensity normalized by z‐score (blue: low abundance; red: high abundance). [file PCE-49-398-s001.pptx]

## Slide 1
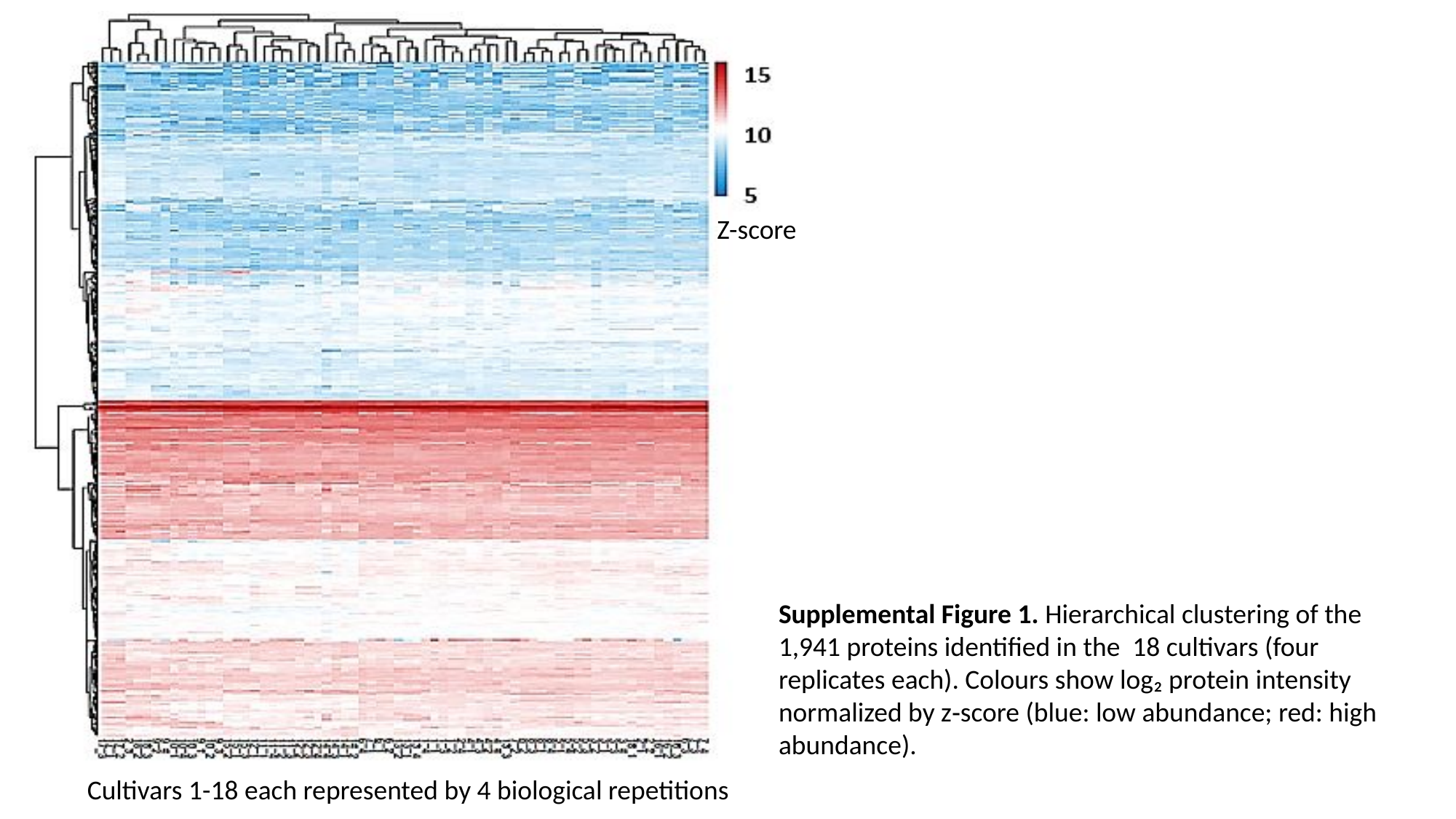

Z-score
Supplemental Figure 1. Hierarchical clustering of the 1,941 proteins identified in the 18 cultivars (four replicates each). Colours show log₂ protein intensity normalized by z‑score (blue: low abundance; red: high abundance).
Cultivars 1-18 each represented by 4 biological repetitions
