# Supplementary material for: Seed‐Borne Spirosoma pollinicola in Commercial Hazelnuts: A Global Survey of Microbial Presence and Allergen Diversity
Source: Plant Cell Environ. 2025 Oct 6;49(1):398–409. doi: 10.1111/pce.70225 (PMC12675980; doi:10.1111/pce.70225)
Supplement: Supplementary file 2 — Supplemental Figure 2: Functional enrichment analysis of proteins identified in the whole proteome. Gene Ontology (GO) terms for Molecular Function (MF), Biological Process (BP), and Cellular Component (CC). After filtering based on protein intensity, consistency across replicates, and GO annotation availability, 1,402 proteins were included in GO enrichment analysis. A total of 424, 809, and 169 GO terms are presented for MF, BP, and CC, respectively. Functional enrichment was performed using g:Profiler (https://biit.cs.ut.ee/gprofiler/). Each filled circle represents a specific GO term within the respective category. Numbers associated with the dots indicate the top enriched GO terms which are listed in Supplemental Table 3. P values were adjusted for multiple testing using the Benjamini‐Hochberg method (Padj). [file PCE-49-398-s006.pptx]

## Slide 1
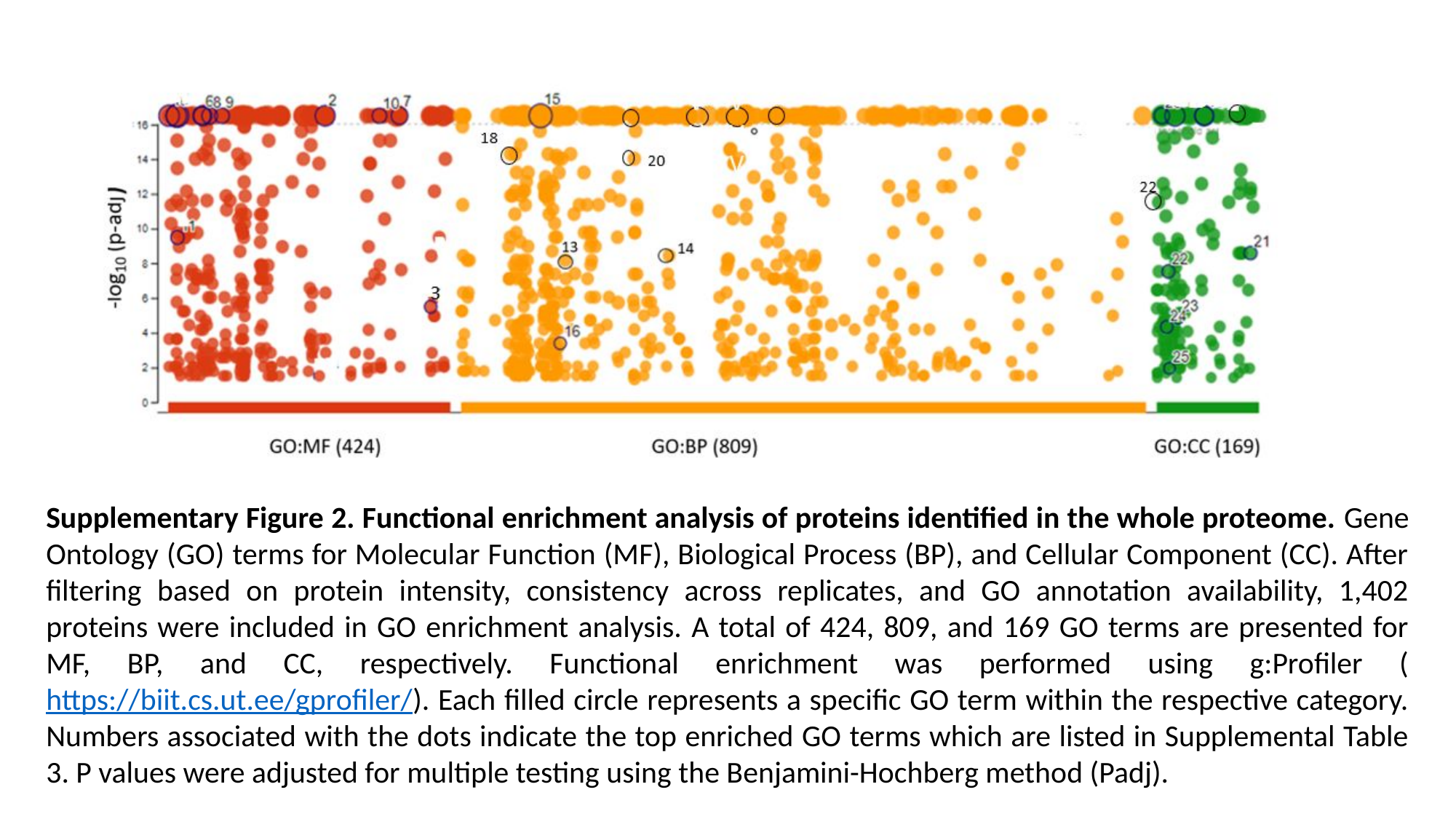

Supplementary Figure 2. Functional enrichment analysis of proteins identified in the whole proteome. Gene Ontology (GO) terms for Molecular Function (MF), Biological Process (BP), and Cellular Component (CC). After filtering based on protein intensity, consistency across replicates, and GO annotation availability, 1,402 proteins were included in GO enrichment analysis. A total of 424, 809, and 169 GO terms are presented for MF, BP, and CC, respectively. Functional enrichment was performed using g:Profiler (https://biit.cs.ut.ee/gprofiler/). Each filled circle represents a specific GO term within the respective category. Numbers associated with the dots indicate the top enriched GO terms which are listed in Supplemental Table 3. P values were adjusted for multiple testing using the Benjamini-Hochberg method (Padj).
