# Supplementary material for: Seed‐Borne Spirosoma pollinicola in Commercial Hazelnuts: A Global Survey of Microbial Presence and Allergen Diversity
Source: Plant Cell Environ. 2025 Oct 6;49(1):398–409. doi: 10.1111/pce.70225 (PMC12675980; doi:10.1111/pce.70225)
Supplement: Supplementary file 3 — Supplemental Figure 3: Gene Ontology (A, GO) function enrichment analysis of the hazelnut proteome, in which 371 proteins had statistically significant changes in protein abundance. GO categories: molecular function (MF), biological processes (BP) and cellular component (CC). The analysis included 371 differentially expressed proteins. X axis represents GO function (three categories: BP‐biological processes, MF‐ molecular function, CC‐ cellular components). [file PCE-49-398-s003.pptx]

## Slide 1
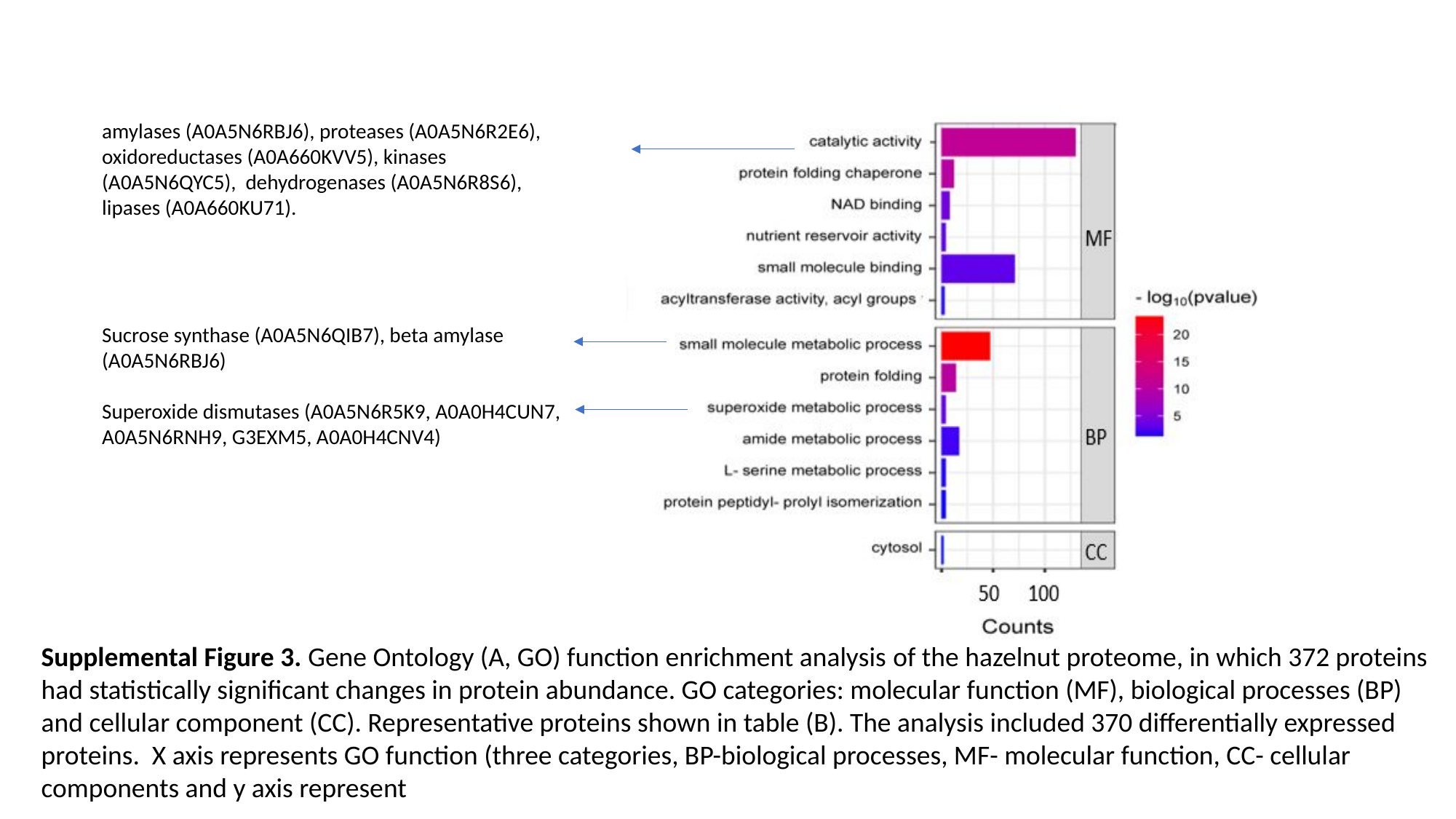

amylases (A0A5N6RBJ6), proteases (A0A5N6R2E6), oxidoreductases (A0A660KVV5), kinases (A0A5N6QYC5), dehydrogenases (A0A5N6R8S6), lipases (A0A660KU71).
Sucrose synthase (A0A5N6QIB7), beta amylase (A0A5N6RBJ6)
Superoxide dismutases (A0A5N6R5K9, A0A0H4CUN7, A0A5N6RNH9, G3EXM5, A0A0H4CNV4)
Supplemental Figure 3. Gene Ontology (A, GO) function enrichment analysis of the hazelnut proteome, in which 372 proteins had statistically significant changes in protein abundance. GO categories: molecular function (MF), biological processes (BP) and cellular component (CC). Representative proteins shown in table (B). The analysis included 370 differentially expressed proteins. X axis represents GO function (three categories, BP-biological processes, MF- molecular function, CC- cellular components and y axis represent
